# Potential Role of Myeloid-Derived Suppressor Cells (MDSCs) in Age-Related Macular Degeneration (AMD)

**DOI:** 10.3389/fimmu.2020.00384

**Published:** 2020-03-20

**Authors:** Anu Kauppinen, Kai Kaarniranta, Antero Salminen

**Affiliations:** ^1^Faculty of Health Sciences, School of Pharmacy, University of Eastern Finland, Kuopio, Finland; ^2^Department of Ophthalmology, Institute of Clinical Medicine, University of Eastern Finland, Kuopio, Finland; ^3^Department of Ophthalmology, Kuopio University Hospital, Kuopio, Finland; ^4^Department of Neurology, Institute of Clinical Medicine, University of Eastern Finland, Kuopio, Finland

**Keywords:** myeloid-derived suppressor cell, age-related macular degeneration, inflammation, innate immunity, adaptive immunity

## Abstract

Myeloid cells, such as granulocytes/neutrophils and macrophages, have responsibilities that include pathogen destruction, waste material degradation, or antigen presentation upon inflammation. During persistent stress, myeloid cells can remain partially differentiated and adopt immunosuppressive functions. Myeloid-derived suppressor cells (MDSCs) are primarily beneficial upon restoring homeostasis after inflammation. Because of their ability to suppress adaptive immunity, MDSCs can also ameliorate autoimmune diseases and semi-allogenic responses, e.g., in pregnancy or transplantation. However, immunosuppression is not always desirable. In certain conditions, such as cancer or chronically inflamed tissue, MDSCs prevent restorative immune responses and thereby aggravate disease progression. Age-related macular degeneration (AMD) is the most common disease in Western countries that severely threatens the central vision of aged people. The pathogenesis of this multifactorial disease is not fully elucidated, but inflammation is known to participate in both dry and wet AMD. In this paper, we provide an overview about the potential role of MDSCs in the pathogenesis of AMD.

## Introduction

Hematopoietic stem cells (HSCs) produce lymphoid and myeloid blood cells. Lymphoid cells include T and B lymphocytes and natural killer (NK) cells, whereas myeloid cells include monocytes, macrophages, granulocytes, erythrocytes, megakaryocytes, and platelets (1). Despite their roles in the innate immune system, myeloid cells can also function as suppressors, although that task has more traditionally been associated with regulatory T cells. Myeloid-derived suppressor cells (MDSCs) remain immature and are a phenotypically and functionally heterogeneous cell population with immunosuppression as their common denominator. MDSCs are widely known for their capacity to suppress host T cell responses against tumor tissue, but they can also be generated upon other stressful conditions, from infections to autoimmunity and obesity, and suppress other cell types, including dendritic cells, NK cells, or macrophages (2–5). MDSCs are not uncommon in ocular diseases either, and they have been studied especially in experimental autoimmune uveitis, a murine model of posterior uveitis of autoimmune origin where retina-specific T cells promote local inflammation, leading to the breakdown of the blood—retinal barrier (BRB), as well as retinal granulomas, folding, and detachment (6, 7). Monocytic MDSCs have also been shown to protect retinal ganglion cells from glutamate-induced damage (8). Despite the potential of MDSCs to enter the retina, their role in other retinal diseases, such as age-related macular degeneration (AMD), has remained elusive.

## Pathophysiology of AMD

**Figure 1:**
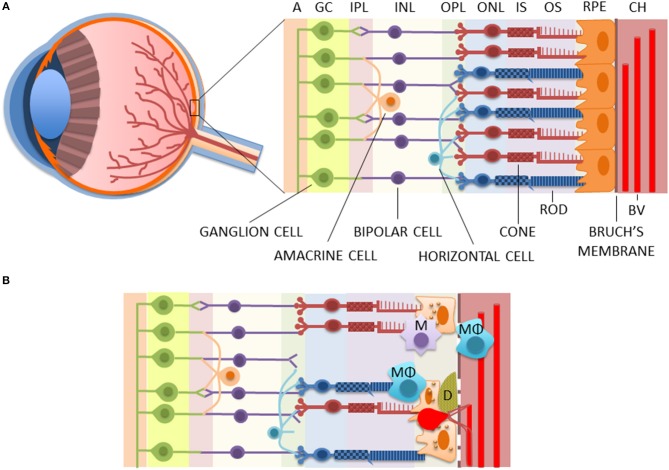
The structure of healthy **(A)** and diseased **(B)** retinae. AMD is associated with the death of RPE and photoreceptors, rupturing of the Bruch's membrane, the accumulation of macrophages and microglia in the choroidea and/or subretinal area, and the deposition of drusen between the RPE and the Bruch's membrane, as well as the accumulation of lipofuscin inside the RPE cells. Moreover, in wet AMD, fragile blood vessels sprout from the choroidea in a process called choroidal neovascularization (CNV) to the retina where they leak causing edema and rapid vision loss. A, axons of optic nerves; BV, blood vessel; CH, the choroidea; D, drusen; GC, ganglion cells; INL, inner nuclear layer; IPL, inner plexiform layer; IS, inner segments of photoreceptors; M, microglia; MΦ, macrophage; ONL, outer nuclear layer; OPL, outer plexiform layer; OS, outer segments of photoreceptors; RPE, retinal pigment epithelium cells.

AMD is the leading cause of severe vision loss among the elderly in developed countries (9). Prolonged life expectancies further amplify its prevalence and cause a vast economic and national health burden. AMD disturbs central vision due to the loss of photoreceptors in the macula, a photoreceptor-dense retinal area responsible for the fine visual acuity (Figure 1) (10). There are two forms of the disease; advanced retinal atrophy is known as dry AMD, and it slowly disturbs the central vision (11). In wet (also known as exudative or neovascular) AMD, which comprises ca. 10–15% of the cases, fragile blood vessels sprout from the choroid into the retina (11). Those neovessels rupture easily and cause edema and acute vision loss (12). It is commonly believed that the disease begins as a slowly progressing dry form that later in some people converts into wet AMD but mechanisms have remained elusive. In a recent study, Krogh-Nielsen et al. suggested that difference between the two disease forms could be associated with age and AMD pathology-related changes in the structure and the functionality of the Bruch's membrane. They showed a positive correlation in dry AMD patients between age and the expression of tissue inhibitor of metalloproteinase (TIMP)-1, a regulator of matrix metalloproteinases (MMPs) with anti-angiogenic properties (13). Conversely, plasma levels of TIMP-3/MMP-2 ratios were significantly lower in patients with wet AMD (13).

As an age-related disease, changes resulting in clinical AMD accumulate over years, even decades. Normal aging contributes to retinal alterations, such as photoreceptor loss, Bruch's membrane thickening, choroid thinning, and formation of hard drusen in the retinal periphery; but in AMD, these become emphasized (11). In addition, AMD involves soft drusen formation at the macular area. Yellowish drusen are extracellular deposits of cellular debris, lipids, lipoproteins, amyloid deposits, and various proteins between the retinal pigment epithelium (RPE) and the Bruch's membrane (Figure 1) (11). They are typically among the first clinical signs upon diagnosis of AMD (14). Drusen-related proteins include various immune system-associated factors, such as complement components, and AMD-related soft drusen are also highly immunoreactive (11). Drusen material is known, for example, to activate inflammasome signaling in RPE cells and macrophage infiltration in the diseased retina (15–18).

The single-cell layer of retinal pigment epithelium (RPE) plays a significant role in the pathogenesis of AMD, and its degeneration is preceding the photoreceptor death in both dry and wet AMD (12, 19). In normal conditions, one major task of RPE cells is to phagocytize spent tips of photoreceptor outer segments (POS) and degrade them by autophagy (19). Efficient removal of waste material is critical, since ca. 10% of the photoreceptor layer volume becomes shed and degraded every day (20). However, aging deteriorates the functionality of intracellular degradation systems, which results in the accumulation of waste products in postmitotic and metabolically active RPE cells (19, 21). This results in increased oxidative stress and accumulation of non-degradable lipofuscin in lysosomes, which are responsible for waste removal by their enzymes (19). Lipofuscin inhibits autophagy, leading to the accumulation of aged mitochondria, which is one of the established characters of AMD (22, 23). In normal conditions, defective mitochondria become degraded by autophagy in a process called mitophagy; but upon autophagy blockade, they continue producing excessive amounts of reactive oxygen species (ROS). Dysfunctional autophagy promotes inflammation in RPE cells through inflammasome activation (24), and oxidative stress is the principal mechanism triggering the activation of NLRP3 receptor responsible for the inflammasome complex assembly (25). The vicious circle between dysfunctional autophagy, mitochondria, and inflammasome activation contributes to the development of pro-inflammatory retinal milieu that further promotes the deposition of lipofuscin and drusen material (19, 19). In addition to local pathology, systemic changes in the levels of certain factors, such as IL-6 or soluble TNF receptor II, have also been associated with AMD (26, 27).

## Role of Myeloid Cells in AMD Pathology

Inflammation is a physiological first-line response to any factor endangering cellular homeostasis (28). Cytokines and chemokines produced in response to pattern-recognition receptor (PRR) activation-induced signaling cascades alert the immune system to restore homeostasis. Chemokines are messengers specialized in attracting leukocytes to the inflamed tissue. In normal conditions, the eye has immune privilege maintained by the BRB, which restricts the infiltration of blood-derived leukocytes, but does not entirely prevent it, especially during aging (29). Microglia are resident inflammatory cells at the retina, which normally locate in the inner layers of the neural retina near to retinal blood vessels (10). Microglia cells play an important role in maintaining homeostasis at the retina where they migrate back and forth to the subretinal space between RPE cells and photoreceptors. In AMD, prolonged existence of stress factors contributes to the tendency of microglia to accumulate in the subretinal space, which aggravates retinal degeneration (10, 30). Relocation of microglia to the subretinal space induces extravasation of myeloid cells through retinal vessels to replace the lack of microglia in the inner retina (31). C-C chemokine receptor type 2-positive (CCR2^+^) monocytes even differentiate into microglia-like cells after arrival (31).

AMD is associated with the rupture of BRB, allowing chemokines to recruit leukocytes also from the underlying choroid and systemic circulation to the retina (10). It has been shown both in dry AMD patients and the *Cryba1* cKO mouse model with conditional knockout of the gene encoding βA3/A1-crystallin that early AMD is associated with infiltration of neutrophils to the choroid and the retina (32, 33). Infiltration of monocytes and their differentiation to macrophages upon retinal damage has been proven by various studies (34–37). Still, the fate of immune cells, especially microglia and monocyte/macrophages upon retinal damage is inadequately known (36). Despite observed leukocyte infiltration in the retina during the development of both AMD forms, it is possible that reduced oxygen consumption due to degeneration of photoreceptors alleviates the attraction of leukocytes in dry AMD. This view is supported by the fact that patients with advanced dry AMD lack significant macular edema or immune cell infiltration (38).

AMD-related leukocyte infiltration can be inflicted by impairment in receptors responding to chemokines that yield an increasing concentration gradient toward the inflamed tissue. C-X3-C Motif Receptor 1 (CX3CR1) and CCR2 are chemokine receptors implicated in drusen formation and the development of AMD (39). Interestingly, monocytes expressing both CX3CR1 and CCR2 receptors have been classified as inflammatory, whereas cells expressing only CX3CR1 have been termed anti-inflammatory (40). CX3CR1 and CCR2 ligands C-X3-C Motif Ligand 1 (CX3CL1 or fractalkine/human, neurotactin/mouse) and Monocyte Chemoattractant Protein 1 (MCP-1 or C-C Motif Chemokine Ligand 2, CCL2), respectively, recruit especially macrophages to inflamed tissue as well as microglia to and from the subretinal space (39, 41). CCL2 is also capable of attracting effector T cells, regulatory T (T reg) cells, and MDSCs (42, 43).

CX3CL1 is a transmembrane protein with integrin-like ability to bind monocytes and T cells, which can also be cleaved into a soluble form with chemotactic capacity (44). Several ocular tissues, including the RPE, constantly expresses CX3CL1 to control the redistribution and activity of CX3CR1-expressing microglia (40, 45). Dysfunctionality or loss of CX3CR1 results in the subretinal accumulation of microglia, which contributes to drusen-like lesions, retinal degeneration, and neovascularization (40). Also, prominent infiltration of inflammatory monocytes in the subretinal space has been associated with photoreceptor death through the P_2_X_7_R-dependent NLRP3 inflammasome activation and IL-1β production in *Cx3cr1*-deficient mice (37, 46, 47). Dysregulated microglia-mediated neurotoxicity upon CX3CR1 deficiency is also known in central nervous system (CNS)-related conditions, such as CNS response to systemic endotoxin-induced inflammation, Parkinson's disease, and amyotrophic lateral sclerosis (ALS) (48). On the other hand, subretinal accumulation of microglia and macrophages also increase during aging irrespective of CX3CR1 expression (49, 50). In a prospective case-control study, higher proportions of CX3CR1^+^ and CCR2^+^ non-classical monocytes were found from peripheral blood of patients suffering from wet AMD when compared to age-matched control subjects devoid of AMD (51). Together, the data suggest that mononuclear cells accumulated at the subretinal space contribute to the retinal degeneration and photoreceptor loss. The role of CX3CR1 in the cell infiltration remains elusive but its increased expression in the peripheral monocytes of wet AMD patients does not exclude disease-specific changes in chemokine receptors.

## Adaptive Immunity in AMD Patients

In the case that acute inflammation cannot be resolved cannot be resolved, the adaptive immune system becomes activated (52). According to current knowledge, in AMD, this refers to T lymphocyte-dependent responses more than B lymphocytes. No differences in the levels of B cells between AMD patients and control subjects have been observed, but oxidative stress-induced neoepitopes and increased concentrations of retinal auto-antibodies in patients with either dry or wet AMD imply that B lymphocytes can be associated with the disease pathogenesis (53–55). In contrast to that, more findings point to the role of T lymphocytes in AMD. T cells can roughly be categorized as helper T (Th) cells, cytotoxic T (Tc) cells, γδ-T cells, and T reg cells (56).

Healthy RPE contributes to the formation of physical BRB and actively removes infiltrating T cells by killing them through Fas-mediated apoptosis or rendering them anergic (57, 58). There is evidence suggesting that in AMD, T lymphocytes can escape elimination or anergization or be functionally altered or impaired from being capable of responding to regulatory signals (52, 59). Also, disease-associated deviations in aging immune system have been reported. For example, increased levels of CD28^−^CD56^+^ T cells in AMD patients point toward an immunosenescent phenotype, as with similar association, found also in coronary artery disease, rheumatoid arthritis, and Behçet's uveitis (60, 61). Moreover, age-dependent reduction in the amounts of Th1 cells was observed in peripheral blood of healthy relatives but not in patients suffering either from dry or wet AMD (62). In a small study by Yu et al., higher levels of IFN-γ and IL-4 were measured from PHA-stimulated PBMC cultures of wet AMD patients in comparison to control subjects, suggesting reactivity of Th1 and Th2 cells in patients (63). The finding on Th1 cells is supported by Chen et al. who showed increased levels of IFN-γ and IL-17-expressing CD4^+^ T cells in the circulation of wet AMD patients when compared to control subjects (64). Th1 and Th17 cells isolated from patients also shifted monocytes toward a pro-inflammatory M1 macrophage phenotype that has been associated with retinal damage (64). In addition to data implying systemic activation of adaptive immunity, in an experimental model of AMD, cytotoxic CD8^+^ T cells directly facilitated RPE degeneration following the immunization of mice with carboxyethylpyrrole (CEP)-modified albumin in complete Freund's adjuvant (65). In another mouse model, laser-induced CNV resulted in the infiltration of IL-17-producing γδ-T cells into the eye, which subsequently promoted inflammation in RPE cells (56). Retinal infiltration of IL-17-producing γδ-T cells was also detected in mice deficient in anti-oxidant system-regulating nuclear erythroid 2-related factor 2 (Nrf2) exposed to a high-fat, cholesterol-rich diet (59). The findings of deleterious effects are contradictory to the role of γδ-T cells as intraepithelial lymphocyte (IEL)-like cells with protective functions upon inflammatory environment and RPE degeneration (66). The outcome is probably related to local conditions, since inflammasome-associated cytokine IL-1β and the alarmin protein high-mobility group box 1 (HMGB1) are capable of promoting IL-17 expression by γδ-T cells (67). Conversely, inhibition of IL-1β and HMGB1 or depletion of γδ-T cells prevented experimental CNV in laser-treated mice (67). Keeping in mind that inflammasome activity and autophagy are inversely dependent on each other, dysfunctional autophagy in aged RPE cells may promote infiltration of IL-17-producing γδ-T cells through inflammasome activation.

## Myeloid-Derived Suppressor Cells

**Figure 2:**
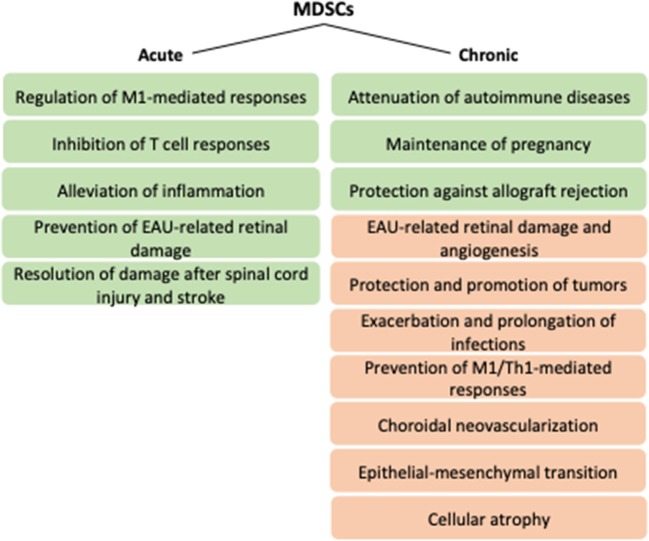
Potential functions of MDSCs upon acute versus chronic conditions. Green and orange boxes refer to positive and negative effects, respectively. EAU, experimental autoimmune uveitis; M1, type 1 macrophage; MDSCs, myeloid-derived suppressor cells; Th1, type 1 helper T cell.

Acute inflammation induces myelopoiesis, strongly expanding neutrophils and monocytes, the latter of which differentiate into macrophages or dendritic cells in tissues depending on local conditions (68, 69). Those cells are active in restoring tissue homeostasis by phagocytosis, respiratory bursts, and promotion of further immune responses by secreting cytokines and activating adaptive immunity (69). Upon persistent stress, myeloid cells remain partially differentiated and adopt immunosuppressive functions (69). MDSCs were initially characterized in mice on their expression of Gr-1 in addition to the classical myeloid marker CD11b (70). Gr-1 is comprised of Ly6C and Ly6G that represent monocytic and granulocytic MDSCs, respectively (71). Due to the lack of homolog for Gr-1, human MDSCs are generally called mononuclear/monocytic (M-MDSC) and polymorphonuclear/granuclocytic (PMN-MDSC) cells (72). M-MDSCs are defined as CD11b^+^CD14^+^HLA-DR^−/lo^CD15^−^ and PMN-MDSCs as CD11b^+^CD14^−^CD15^+^ or CD11b^+^CD14^−^CD66b^+^ (71). Also, the myeloid marker CD33 can be used to define MDSCs; it is expressed by M-MDSCs, whereas PMN-MDSCs display DC33^dim^ staining (71). Lin^−^HLA-DR^−^CD33^+^ are early stage MDSCs that can be found from human, but their equivalent in mice is not known yet (71). Physiologically, MDSCs are advantageous in semi-allogenic situations, such as pregnancy or transplantation, but they are better known for their disadvantageous immunosuppressive functions in chronic pathologies, for example, in chronic infections or cancer (72, 73) (Figure 2). MDSCs can regulate both innate and adaptive immune responses, e.g., by modulating macrophages, inhibiting NK or T cell responses or by inducing regulatory T cells (2, 5, 74–79). Their non-immunological functions include promotion of angiogenesis and metastasis (2, 80). Factors by which MDSCs execute their effects include arginase 1 (Arg-1), indoleamine dioxygenase (IDO), IL-10, inducible nitric oxide synthase (iNOS), nitric oxide (NO), heme oxygenase 1 (HO-1), carbon monoxide (CO), prostaglandin E2 (PGE2), ROS, and cysteine depletion (2, 73). The anergization of NK cells is dependent on the membrane-bound TGF-β1 of MDSCs, and intracellular HO-1 of MDSCs regulates T cell proliferation by CO production (73, 75). HO-1, as a stress-responsive enzyme with immunoregulatory and cytoprotective properties, is capable of protecting against oxidative stress, regulating cell proliferation, modulating inflammatory responses, and facilitating angiogenesis (81). HO-1 has been observed as a central mediator in MDSC-associated suppression upon transplantation (82). Along with the expression of regulatory cytokine IL-10, HMOX-1 encoding for HO-1 was reduced in monocytic MDSCs of secondary progressive multiple sclerosis (MS) patients when compared to relapsing–remitting MS patients or healthy subjects (83). HO-1 also participates in the tumor microenvironment to protect tumor cells from apoptosis, to improve their growth and potentially also metastasis (81, 84). Collectively, MDSCs are beneficial in quenching acute inflammation, autoimmune diseases, and responses against (semi-)allografts but become detrimental upon chronic inflammatory and neoplastic conditions.

## MDSCs in the Maintenance of Retinal Homeostasis

Besides the contribution to physical BRB, RPE cells have also other means to maintain homeostasis at the retina (38). Secretion of TGF-β or thrombospondin-1 (TSP-1) and the expression of programmed death-ligand 1 (PD-L1/CD247/B7-H1) on the plasma membrane alleviate T and B cell responses, and the production of cathepsin L inhibitor CTLA-2α promotes the induction of CD4^+^CD25^+^Foxp3^+^ T reg cells (85–90). RPE cells also produce soluble neuropeptides with immunomodulatory capacities. Kawanaka et al. showed that neuropeptides alpha-melanocyte stimulating factor (α-MSF) and neuropeptide Y (NPY) induced co-expression of Arg1 and NOS2 in resting macrophages, converting them into MDSC-resembling cells that showed significantly reduced secretion of pro-inflammatory cytokines upon exposure to lipopolysaccharide (LPS) (91). In contrast, cells expressing only Arg1 or NOS2 but not both were found from laser-wounded retina (91). Cells lacking co-expression were less efficient in inducing apoptosis in T cells and contributed to the development of pro-inflammatory milieu. As an additional mechanism, RPE cells have been shown capable of inducing MDSC differentiation from co-cultured bone marrow (BM) progenitor cells upon exposure to granulocyte–macrophage colony-stimulating factor (GM-CSF) and IL-4 (7). In the absence of RPE cells, BM cells were differentiated into dendritic cells in the presence of given cytokines, as expected (7). CD11b^+^Gr-1^+^ MDSCs shared similar surface markers with tumor MDSCs, they efficiently inhibited T cell proliferation and inflammatory cytokine production, and their systemic delivery to mice immunized with the interphotoreceptor retinoid-binding protein (IRBP)_1−−20_ peptide in complete Freund's adjuvant (CFA) prevented EAU-related retinal injury (7). The pro-inflammatory cytokine IL-6 was needed for the process since its blockade reduced the RPE cell-induced MDSC differentiation. Instead, TGF-β did not participate in the differentiation process (7). Both IL-6 and TGF-β can be very harmful due to their capacity to induce inflammation and fibrosis, respectively, but probably concentrations and locations influence their effects (86).

## Potential of MDSCs in Provoking Angiogenesis in Diseased Retina

Chronic inflammation is known as a promoter for MDSC differentiation and functionality. MDSCs have also been experimentally induced using different cytokine cocktails (92). Bunt et al. showed in murine cells that IL-1β-induced inflammation activated MDSCs through the TLR4/CD14 pathway making MDSCs to regulate macrophages by increasing their IL-10 and reducing IL-12 productions (93). That may refer to a homeostatic process where M2-type macrophages producing immunosuppressive IL-10 prevent the expansion of M1 population, which by releasing pro-inflammatory cytokines, such as IL-12 and TNF-α, is associated with tissue destruction and T helper 1 (Th1) cell activation (65). On the other hand, anti-inflammatory but pro-angiogenic M2 macrophages have been associated with choroidal neovascularization and the development of wet AMD (94, 95).

Drusen material accumulating between the RPE and the Bruch's membrane diminish the oxygen supply from the choroid to the retina (96). RPE cells suffering from hypoxia can result in the release of vascular endothelial growth factor (VEGF) already prior to the production of inflammatory markers (97). Upon laser-induced vessel formation, peak in the VEGF production coincided with the arrival of macrophages, supporting the link between RPE-associated promotion of neovascularization (98). Macrophages sense the need for oxygen by retinal cells and further potentiate the VEGF-mediated neovessel formation (10). In cancer, inflammation-induced MDSCs inhibit immunosurveillance to allow persistence and proliferation of premalignant and malignant cells (99). The capacity of MDSCs to induce angiogenesis has not been shown only in cancer studies but also in the neovascularization of an ischemic hind-limb of mice (80, 100).

It is intriguing to hypothesize that cyclooxygenase-2 (COX-2) in choroidal neovascular membranes could maintain elevated MDSC levels such that neovessel growth is promoted. COX-2 is the predominant cyclooxygenase in human RPE cells, and inflammatory cytokines further promote its expression (101). COX-2 activity is capable of recruiting MDSCs through CCL2 or PGE2. COX-2 converts arachidonic acid to PGE2 that plays a major role in the physiologic induction of MDSCs (102). Direct effects of PGE2 through the E-prostanoid (EP) receptor provide an alternative pathway for *Ccl-2*- or *Ccr-2*-deficient mice to develop atrophic (dry) and neovascular (wet) pathologies in the absence of the chemokine or its receptor (103). In an *in vitro* study with mouse primary RPE cells, the major lipofuscin component bis-retinoid N-retinylidene-N-retinylethanolamine (A2E) reduced PGE2 levels and promoted RPE cells to induce Th1 cell differentiation in IL-1β-dependent way, which might thereby contribute to further retinal degeneration (104, 105).

COX-2 inhibition by acetylsalisylic acid (aspirin, ASA) prevented the CCL2-mediated accumulation of CD11b^+^Ly6G^hi^Ly6C^lo^ granulocytic MDSCs to the tumor microenviroment in mice with glioma (43). COX-2/CCL2 blockade also increased the expression of C-X-C Motif Chemokine 10 (CXCL10/Interferon γ-induced Protein 10/IP-10) that inhibits VEGF-mediated angiogenesis (43, 52). COX-2 is expressed by human choroidal neovascular membranes (106), and promotion of CXCL10 could result in its inhibition. CXCL10 is a ligand of C-X-C Motif Chemokine Receptor 3 (CXCR3 also known as GPR9 or CD183) that, along with C-C Chemokine Receptor Type 3 (CCR3), is associated with the development of wet AMD (52). Percentage of both CD4^+^ Th and CD8^+^ Tc cells expressing CXCR3 has been observed to be lower in the peripheral blood of patients with wet AMD in comparison to control subjects (62, 107), which may diminish the benefit of increased CXCL10 production following the COX-2 inhibition. Acetylsalisylic acid is a non-steroidal anti-inflammatory drug (NSAID) and COX-2 inhibitor that is commonly used at low doses for long periods due to its anti-thrombotic effects. A retrospective study on AREDS and AREDS2 data supports the inability of COX-2 inhibition to protect from neovascularization since the use of acetylsalisylic acid was not significantly associated with progression of either dry or wet AMD (108). Instead, a prospective double-blind randomized human study on the therapy of wet AMD with photodynamic therapy (PDT) supplemented with oral intake of the COX-2 inhibitor nabumetone resulted in the progression of macular atrophy (109). Collectively, the data on COX-2 inhibition suggest no beneficial effects on wet AMD but potential aggravation in the progression of dry AMD, thereby conflicting with the idea of COX-2-induced and MDSC-mediated neovascularization.

## Complement and MDSCs

Due to facts concerning genetic predisposition and composition of drusen, it is evident that the complement pathway contributes to the pathogenesis of AMD. Excessive complement activation has also been observed in various studies to be harmful in pathologic retinal degenerative and angiogenic conditions (110).

Complement components produced by RPE cells contribute to the formation of sub-RPE deposits through complement-driven proteasome inhibition and release pro-inflammatory cytokines, such as IL-6 and IL-1β, that can amplify the response by further promoting expression of the C3a receptor (111, 112). Unstimulated RPE cells express significantly higher levels of complement regulator genes in comparison to complement component genes but under inflammatory conditions, activated macrophages induce complement factor B (CFB) and C3 expression in RPE cells (113). Laser photocoagulation has also shown to induce the deposition of C3 and membrane attack complex (MAC) in the neovascular complex (114). Furthermore, MAC formation is capable of releasing angiogenesis-related growth factors, such as β-fibroblast growth factor (β-FGF), VEGF, and platelet-derived growth factor (PDGF) (114). The role of MDSCs has not been studied in relation to ocular complement activation but there is evidence on the MDSC contribution from other disease models. Complement inhibition reduces tumor growth with accompanying decrease in the both C3 and MDSC levels (115). Hsieh et al. also showed that C3 plays a major role in the conversion of bone marrow progenitor cells into MDSC by hepatic stellate cells (116, 117). Those MDSCs efficiently inhibited T cell responses *in vitro* and *in vivo*, and alleviated experimental autoimmune myasthenia gravis also by reducing anti-actylcholine receptor IgG levels and decreasing complement activation at the endplates of neuromuscular junctions (116–118).

Active pro-inflammatory complement components C3a and C5a (anaphylatoxins) are included in drusen material and efficiently promote choroidal neovascularization in mice by stimulating VEGF production in RPE and choroid cells (98). Conversely, normalized anaphylatoxin levels promoted recovery from CNV lesions and prevented fibrotic scar formation (119). A study on the ARPE-19 cell line originating from spontaneously transformed human RPE cells emphasized the role of C5a over C3a in the induction of VEGF production, but Nozaki et al. also found C3a to stimulate VEGF production in primary human RPE cells in addition to D407 RPE cell line (120, 121). Both C3a and C5a were found from the drusen, the proximity of RPE cells, and the Bruch's membrane of an AMD patient, whereas neither of them were present in the eye of a control subject without AMD (98). A study on C3aR^−/−^ and C5aR^−/−^ mice showed that complement components also play a role in the recruitment of neutrophils and macrophages that were observed to peak one and three days after the insult, respectively (98). Neutrophils further pave the way to other leukocytes by expressing MMP-9 that disrupts the integrity of BRB (122). In dry AMD, photoreceptor outer segments exposed to human serum following the BRB breakdown resulted in complement activation, C5a-mediated attraction of peripheral blood monocytes, and diminished survival of the RPE and neural retina (123). Complement activation in subretinal macrophages can potentially play a role in the pathogenesis of AMD (124). Also systemic complement activation in AMD patients has been shown (125, 126).

Substitution of histidine to tyrosine (Y402H) is one of the best-known polymorphic complement factor H (CFH) variants associated with AMD. By diminishing the functionality of the regulatory CFH, it results in overactive alternative complement pathway (10). Incomplete CFH activity has been shown to enhance C3 deposition and C5a release in non-small lung cancer cell lines (127–130). In addition to direct receptor binding, C5a can stimulate MDSCs also indirectly through IL-6, IL-1β, or VEGF (131). The role of C5a in cancer promotion is well known, and it is also considered as a key player in poor cancer prognosis on subjects suffering from an autoimmune disease with complement activation by immune complexes (the Arthus reaction) (132). According to a recent discovery, CFH(Y402H) also efficiently binds CD11b, preventing CD47-mediated elimination of mononuclear cells, such as macrophages and microglia, attracted to the subretinal space between the RPE and photoreceptor outer segments to restore homeostasis (133, 134). This may extend the presence of mononuclear cells at the retina beyond the need for homeostasis restoration, which predisposes to adverse effects, such as photoreceptor degeneration and choroidal neovascularization (41, 135, 136). CD11b is also expressed by both monocytic and granulocytic MDSCs (71) predisposing the retina to their prolonged impact in susceptible persons. Overall, complement and CFH(Y402H) are efficient in activating MDSC and may thereby prevent the removal of drusen material by immune cells.

## MDSCs Enhance Fibrosis

Choroidal neovascularization does not only cause rapid vision loss due to bleeding and swelling at the retina, but it also results in subretinal fibrosis that can develop despite the anti-VEGF therapy that prevents the neovessel formation (137). Conversely, epithelial-mesenchymal transition (EMT) can also cause resistance to anti-VEGF drugs, as suggested by studies on pancreatic cancer cells (138). Subretinal fibrosis is associated with the end stage pathogenesis of wet AMD (137). TGF-β signaling in myeloid cells promotes CCL2-dependent MDSC recruitment to the tumor microenvironment, and granulocytic MDSC-derived TGF-β promotes EMT upon metastasis, which is the advanced form of malignancy (139, 140). TGF-β is a multifunctional growth factor physiologically needed for the development and tissue repair, but excessive concentrations are associated with inflammation and tissue fibrosis, with detrimental consequences in the eye (141, 142).

## Concluding Remarks

MDSCs have been found beneficial in various CNS disorders and autoimmune diseases (Figure 2). Both monocytic and granulocytic MDSCs have been shown to alleviate EAE (143–145). Monocytic MDSCs participated in resolving damage following experimental spinal cord injury in mice, alleviated inflammation after stroke in mice, and were found in increased numbers in the peripheral blood of ischemic stroke patients (146, 147). MDSCs induced by hepatic stellate cells efficiently inhibited T cell responses *in vitro* and *in vivo* and attenuated experimental autoimmune myasthenia gravis also by reducing anti-actylcholine receptor IgG levels and decreasing complement activation at the endplates of neuromuscular junctions (116–118). MDSCs were observed beneficial also especially at the beginning of autoimmune uveoretinitis (6, 148–150). Adoptive transfer of RPE-induced MDSCs reduced EAU severity when detected 21 days after immunization when the acute phase peaks (7). However, upon combined deletion of CCL2 and CX3CR1, deficiency in macrophages and MDSCs during chronic EAU (90 days post-immunization) contributed to reduced retinal damage and angiogenesis (150). The data supports the overall idea of inflammation where the timing matters. In the acute phase, MDSCs probably participate in the restoration of homeostasis, but upon chronic inflammation, their capacity to promote atrophy and angiogenesis may overcome the benefits.

## Author Contributions

AK contributed to the planning of the content, writing of the manuscript, and preparing the figures. AS and KK contributed to the planning of the content and the critical evaluation of the text.

### Conflict of Interest

The authors declare that the research was conducted in the absence of any commercial or financial relationships that could be construed as a potential conflict of interest.
